# Fast and precise pathogen detection and identification of overlapping infection in patients with CUTI based on metagenomic next-generation sequencing: A case report

**DOI:** 10.1097/MD.0000000000027902

**Published:** 2021-12-10

**Authors:** Manyu Zhang, Weixiu Wang, Xue Li, Xiaoxiao Zhang, Dingwei Yang

**Affiliations:** Department of Nephrology, Tianjin Hospital, Tianjin, China.

**Keywords:** case report, chronic renal failure, complicated urinary tract infection, metagenomic next-generation sequencing, overlapping infection

## Abstract

**Rationale:**

The gold standard for pathogen detection and identification of complicated urinary tract infection (CUTI) remains quantitative urine culture, however, the diagnostic value of urine culture remains limited due to the time-consuming procedure and low detection rate. Here we report a case of successfully using Metagenomic next-generation sequencing (mNGS) provided fast and precise detection and identification of overlapping infection in patients with CUTI with no similar reports or studies published before to the best of our knowledge.

**Patient concerns:**

A 70-year-old male was admitted to hospital due to elevated serum creatinine for 2 weeks.

**Diagnoses:**

Acute exacerbation of chronic renal failure and CUTI were the most critical diagnosis on admission.

**Interventions:**

Blood purification, bladder irrigation and aggressive anti-infective therapy were administered. But the empirical anti-infection therapy and the adjustment of treatment according to the evidence of urine culture drug sensitivity had no obvious effect. We further carried out urinary PMseq-DNA detection and the results showed overlapping infection with Enterococcus faecium, Enterococcus hirae, Pseudomonas aeruginosa, Pseudomonas denitrificans and Candida albicans. According to the genetic test results, linezolid, meropenem and fluconazole triple anti-infection treatment was given.

**Outcomes:**

After adjusting the treatment, the infection was basically controlled in 10 days, and even the renal function was significantly improved, dialysis independence was achieved after 3 months.

**Lessons:**

Our case illustrated the potential application of mNGS in detecting pathogenic microorganisms in patients with CUTI especially when overlapping infections are present.

## 1 Introduction

Complicated urinary tract infection (CUTI) is significantly different from general urinary tract infection, mainly manifested as recurrent, persistent, and refractary. The main pathogenic bacteria of CUTI are Gram-negative bacilli, among which Escherichia coli, Klebsiella pneumoniae, and Proteus mirabilis are the most common.^[1]^ However, the number of cases caused by other bacteria is increasing due to the recent serious antibiotic abuse, what followed is pathogen resistance to antibiotics has been increased dramatically, and the risk of sepsis is higher. Incapability of timely pathogen detection and identification might lead to inappropriate treatment, antibiotic resistance, and increased medical costs even poor prognosis. Nevertheless, the gold standard for pathogen detection and identification of urinary tract infection remains quantitative urine culture, which usually takes at least 18 h lead to difficulty in the diagnosis 24 to 48 h after onset.^[2,3]^

Metagenomic next-generation sequencing (mNGS) is a high-throughput sequencing approach with high efficiency and short turnaround time. Pathogen detection and identification by mNGS is an emerging clinical practice, which only needs small amounts of DNA extracted from the sample then could detects and identifies pathogens simultaneously.^[4]^ The technique is culture-independent, and more sensitive than conventional cultures, as it does not rely on an appropriate environment for microbial growth and thus may detect slow-growing, anaerobic, and fastidious organisms.^[5]^ mNGS has already been successfully applied in several clinical trials for infection diagnosis, which provided fast and precise pathogen detection and identification,^[4,6–8]^ however, there remains minimal evidence to support the use of mNGS for the diagnosis or targeted therapy in CUTI.

## 2 Case presentation

A 70-year-old male was admitted to hospital due to elevated serum creatinine for 2 weeks. Two weeks before admission, the patient underwent cystostomy because of urinary retention. Preoperative examination revealed that the serum creatinine increased to 251.8 μmol/L, and the creatinine was rechecked before discharge showed significant increase to 435 μmol/L. The drainage urine was turbid brown with precipitate after cystostomy, without fever, chills, and other special discomfort. Since the onset of the disease, urine volume maintained at 1000 to 1500 mL/day with no obvious abnormality. The patient had a history of myelopathy and high paraplegia for 25 years. Denied the history of hypertension, diabetes, coronary heart disease, cerebrovascular disease and other chronic diseases.

The physical examination on admission was as follows: *T*: 36.30°C *P*: 92 beats/min *R*: 18 times/min Bp: 120/79 mm Hg. The patient had an anemic appearance; coarse breath sounds were heard in both lungs with no obvious rales. No tenderness, rebound tenderness or muscle tension was detected in the whole abdomen, mobile dullness (−), percussive pain in both kidneys (−). The muscle strength of both lower limbs was grade 0, and the muscle strength of both upper limbs was grade 5. There was no obvious edema on the face or limbs. A bladder fistula indwresting was found in the lower abdomen. Laboratory examination after admission is shown in Table 1 and Table 2. And the urologic ultrasound indicated diffuse lesions of both renal parenchyma (left kidney size 12.3 cm × 5.2 cm, parenchyma thickness 1.8 cm, right kidney size 1.4 cm × 5.4 cm, parenchyma thickness 1.5 cm), renal pelvis separation of the right kidney (0.9 cm), and cystostomy post-operative changes. Rheumatic immunity, anti-nuclear antibody, antineutrophil cytoplasmic antibody (ANCA), anti-glomerular basement membrane (GBM) autoantibody, tumor markers, hepatitis B antigen-antibody, and other secondary factors of renal damage screening all returned negative.

**Table 1:** Blood test results (The normal reference range are presented in footnotes^∗^.).

Serum creatinine (CREA-S, μmol/L)	563	Neutrophil (NEU#, ×10^9^/L)	5.28	Calcium (Ca, mmol/L)	2.16
Urea (mmol/L)	26.48	Neutrophil (NEU%, %)	72.20	Phosphorus (P, mmol/L)	2.23
Uric acid (UA, μmol/L)	470	Hemoglobin (HGB, g/L)	79	Parathormone (PTH, pg/mL)	425.1
White blood cell (WBC, ×10^9^/L)	7.31	Platelet (PLT, ×10^9^/L)	221		

**Table 2:** Urine test results (The normal reference range are presented in footnotes^∗^.).

N-acety-ß glucosidase (NAG, U/L)	16.4	Urinary red blood cell	659/HPF
Neutrophil gelatinase-associated lipocalin (NGAL, ng/mL)	1960.5	Urinary red blood cell	3659/μL (Glomerular red blood cells accounted for 60% and non-glomerular red blood cells for 40% with numerous bacteria)
24 h-urinary protein (PRO)	2.893 g (medium and large molecular weight proteins are dominant among them)	Urine culture	Candida albicans (5–10 × 10^4^ CFU/mL)
Urinary WBC	311/HPF	Urinary WBC	1730/μL

The patient was preliminarily diagnosed as acute exacerbation of chronic renal failure, pyelonephritis, renal anemia, hypocalcemia, hyperphosphatemia, and secondary hyperparathyroidism. Bladder irrigation and aggressive anti-infective therapy were administered immediately after admission. Combined with the history of urinary retention, we considered the possibility of acute exacerbation of chronic renal failure induced by obstruction and infection. As a result, conservative treatment was performed for 3 days, and the reexamination results indicated: CREA-S 726 μmol/L, UA 464 μmol/L, UREA 34.60 mmol/L, WBC 9.98 × 10^9^/L, NEU# 6.75 × 10^9^/L, NEU% 67.70%, HGB 73 g/L, PLT 215 × 10^9^/L. Considering the rapid elevation of serum creatinine and the occurrence of nausea, vomiting, and poor appetite, the patient was inserted an internal jugular vein temporary catheter and received blood purification treatment. The gastrointestinal symptoms improved significantly after 3 rounds of regular hemodialysis, and creatinine fluctuation in 336 to 514 μmol/L was detected.

During the treatment, the patient presented intermittent mild temperature rise, fluctuating between 37.1°C and 37.3°C, and the bladder fistula drainage was persistently epinephelos with sediment. Cefodizime was given as empirical anti-infection treatment, then repeated urine culture was examined, Candida Albicans was suggested for many times. On the basis of cefodizime, fluconazole was used for antifungal therapy, but no significant relief was found, reexamined urine routine showed: urinary WBC 8204 to 12,379/μL, urinary red blood cell RBC 1615 to 4032/μL (mainly non-glomerular red blood cells).

Considering that CUTI in the patient was difficult to control, we further carried out urinary PMseq-DNA detection, it took 24 h, and the results showed overlapping infection with Enterococcus faecium (84,273 gene sequences), Enterococcus hirae (2462 gene sequences), Pseudomonas aeruginosa (2444 gene sequences), Pseudomonas denitrificans (39 gene sequences), and Candida albicans (16,849 gene sequences). According to the genetic test results, the patient had overlapping infection of Enterococcus, Pseudomonas aeruginosa, and fungal. With the abuse of antibiotics and the implementation of invasive tests, the bacterial drug resistance becoming increasingly prominent. Fortunately, the sensitivity of Enterococcus to vancomycin and linezolid can reach 80%, drug resistance of Pseudomonas aeruginosa to carbapenems is also <20%, considering the renal toxicity of vancomycin, we chose linezolid for this patient, then linezolid, meropenem and fluconazole triple anti-infection treatment was given. And the therapeutic effect was rapid and dramatic: body temperature was controlled after only 2 days and urine was clear after 4 days. We rechecked urine routine: urinary WBC 359 to 2096/μL, urinary red blood cell RBC 897 to 2296/μL (mainly non-glomerular red blood cells). The infection was basically controlled in 10 days, and even the renal function was improved (we rechecked renal function 3 days after dialysis suspension which indicated that CREA-S 286 μmol/L, UA 231 μmol/L, UREA 12.09 mmol/L). Depending on the patient's condition, the frequency of dialysis was reduced to 1 to 2 times a week. The patient was discharged after 20 days of mNGS and modified anti-infective therapy. Up to now, the patient's condition is stable, with body temperature controlled, clear urine color, urine volume maintained 1500 to 3000 mL/day and renal function significantly improved, dialysis independence was achieved after 3 months.

## 3 Discussion/conclusion

The gold standard for diagnosis of urinary tract infection remains quantitative urine culture, however, the diagnostic value of urine culture remains limited due to the time-consuming procedure and low detection rate, especially in patients who have already used antibiotics. At present, mNGS has been reported to be used in the diagnosis and pathogen identification of infectious diseases in multiple systems, including the central nervous system, circulatory system, respiratory system, digestive system, urinary system, and ocular infections. Initial applications of mNGS for diagnostics focused on central nervous system infections, mostly chronic infections, a single report of the accuracy of mNGS observed a diagnostic sensitivity of 84.3% and specificity of 93.7% compared to standard methods.^[9]^ In terms of urinary tract infections, the case of Li et al illustrated the potential application of mNGS in detecting pathogenic microorganisms in samples which were not detected by traditional culture and serological testing.^[10]^ Ishihara et al observed and studied 10 patients with acute urinary tract infection, concluded that patients who have continued symptoms of acute cystitis despite treatment and with negative urine culture results may benefit from DNA mNGS.^[11]^ In those patients with suspected atypical or anaerobic bacteria, DNA mNGS may also prove more beneficial in diagnosing a UTI. DNA mNGS can help to evaluate for low bacterial loads or resistant infections not otherwise diagnosed with traditional methods.^[12,13]^ On the whole, there are now multiple case reports in which viruses, bacteria, fungi and parasites have been identified from mNGS of cerebrospinal fluid, brain tissue, plasma, sputum, bronchoalveolar fluid, stool, urine and eye specimens, however, experience with mNGS in the diagnosis of multiple overlapping infections is still lacking, for patients with complicated urinary tract overlapping infection, the sensitivity and clinical directivity of mNGS is still unclear. On the other hand, a key disadvantage inherent to mNGS is that microbial nucleic acids from most patients’ samples are dominated by human host background, thus limiting the overall analytical sensitivity of the approach for pathogen detection. Another potential drawback of mNGS is the detection of microbial contaminants present in the sample, reagents used for processing, or laboratory environment, which can complicate the analysis and interpretation of results.^[14]^

In this case, there existed urinary obstruction and bladder fistula in the patient, secondary infection led to rapid deterioration of renal function, and complicated urinary tract led to the possibility of overlapping infection. Although repeated urine culture suggested Candida Albicans, after treatment with fluconazole based on drug sensitivity, body temperature was not completely controlled, and urine color continued to deepen with cloudy precipitation. Therefore, we considered that the sensitivity of routine urine culture to overlapping infection was not strong, and the possibility of infection with mycobacterium tuberculosis, virus and atypical pathogens also could not be ruled out. At the same time, the patient had irregular use of antibiotics before admission, and the empirical use of cefodizime for anti-infection treatment after admission further reduced the positive rate of urine culture. In addition, for typical urine cultures, many laboratories define 10^5^CFU/mL urine as the threshold, however, this threshold misses many relevant infections.^[15]^ Through mNGS, we successfully detected possible pathogens of complicated urinary tract overlapping infection within 24 h which was urine culture undetectable, indicated that the speed and detectability of mNGS were both superior to the traditional quantitative urine culture. The mNGS-detectable, unculturable bacteria included Enterococcus faecium, Enterococcus hirae, Pseudomonas aeruginosa and Pseudomonas denitrificans. The first two belong to the normal intestinal flora, and it is known that E faecalis can be identified in about 80% of human infections.^[16]^ Pseudomonas aeruginosa is widely distributed in nature, human skin, intestinal tract and respiratory tract which is also listed as one of the common opportunistic pathogens. The above three bacteria may cause urinary tract infection, especially in patients with complicated urinary tract. These 3 kinds of mNGS-detectable, unculturable bacteria might be sensitive to third generation cephalosporins, whose DNA was detected by NGS from its dead remnants. Conversely, the use of antibiotics resulted in reduction in pathogenic bacteria load, and the culture results were false-negative due to the residual pathogens were below the threshold of the normal urine culture test. Therefore, urine mNGS tests may be able to detect bacteria even after antibiotic administration which is significantly stronger than traditional culture.^[17]^ But it's important to point out that one common disadvantage of urine evaluated by both culture and mNGS techniques is contamination, contamination of urine culture ranges from 0.8% to 41.7% based on review of current laboratory practices, whereas the median institution in the United States has a contamination rate of 15.0%.^[18]^ In clinical practice, we exclude contaminated microorganisms by setting detection thresholds, the culture reports in our study were quantified as 10^4^ to 10^7^ while DNA mNGS reports on bacterial loads as 10^3^ to 10^4^, as a result, Pseudomonas denitrificans with the least gene sequences detected were judged as contamination. Different criteria may influence the interpretation of the results and the comparison of sensitivities, nevertheless, in this case, the body temperature was controlled 2 days after the adjustment of treatment based on mNGS, urine color was clear in 4 days, renal function was improved in 10 days, and even achieved dialysis independence at 3 months, all of which indicated that the anti-infection program guided by mNGS had significant and rapid effect.

In conclusion, with clinical effect as the gold standard, our case illustrated the potential application of mNGS in detecting pathogenic microorganisms in patients with complicated urinary tract especially when overlapping infections are present. Clinical trials are needed to determine the sensitivity and specificity of mNGS in CUTI.

## Author contributions

**Conceptualization:** Dingwei Yang.

**Data curation:** Manyu Zhang.

**Formal analysis:** Manyu Zhang.

**Investigation:** Xue Li, Xiaoxiao Zhang.

**Methodology:** Weixiu Wang.

**Project administration:** Weixiu Wang, Xue Li, Xiaoxiao Zhang.

**Validation:** Weixiu Wang.

**Writing – original draft:** Manyu Zhang, Weixiu Wang.

**Writing – review & editing:** Dingwei Yang.
